# Down-Regulation of the Canonical Wnt β-Catenin Pathway in the Airway Epithelium of Healthy Smokers and Smokers with COPD

**DOI:** 10.1371/journal.pone.0014793

**Published:** 2011-04-07

**Authors:** Rui Wang, Joumana Ahmed, Guoqing Wang, Ibrahim Hassan, Yael Strulovici-Barel, Neil R. Hackett, Ronald G. Crystal

**Affiliations:** Department of Genetic Medicine, Weill Cornell Medical College, New York, New York, United States of America; Comprehensive Pneumology Center, Germany

## Abstract

**Background:**

The Wnt pathway mediates differentiation of epithelial tissues; depending on the tissue types, Wnt can either drive or inhibit the differentiation process. We hypothesized that key genes in the Wnt pathway are suppressed in the human airway epithelium under the stress of cigarette smoking, a stress associated with dysregulation of the epithelial differentiated state.

**Methodology/Principal Findings:**

Microarrays were used to assess the expression of Wnt-related genes in the small airway epithelium (SAE) obtained via bronchoscopy and brushing of healthy nonsmokers, healthy smokers, and smokers with COPD. Thirty-three of 56 known Wnt-related genes were expressed in the SAE. Wnt pathway downstream mediators β-catenin and the transcription factor 7-like 1 were down-regulated in healthy smokers and smokers with COPD, as were many Wnt target genes. Among the extracellular regulators that suppress the Wnt pathway, secreted frizzled-related protein 2 (SFRP2), was up-regulated 4.3-fold in healthy smokers and 4.9-fold in COPD smokers, an observation confirmed by TaqMan Real-time PCR, Western analysis and immunohistochemistry. Finally, cigarette smoke extract mediated up-regulation of SFRP2 and down-regulation of Wnt target genes in airway epithelial cells *in vitro*.

**Conclusions/Significance:**

Smoking down-regulates the Wnt pathway in the human airway epithelium. In the context that Wnt pathway plays an important role in differentiation of epithelial tissues, the down-regulation of Wnt pathway may contribute to the dysregulation of airway epithelium differentiation observed in smoking-related airway disorders.

## Introduction

The human airway epithelium, a pseudostratified layer of cells derived from the endoderm, serves as a physical barrier against inhaled pathogens, xenobiotics, and other noxious substances in the environment [1], [2]. The normal airway epithelium has the capacity of regeneration and repair [3]–[7]. Of the major cell types, the airway epithelial progenitor cells are in the basal cell population, cells capable of self renewal and differentiation into columnar cells and eventually, the differentiated secretory and ciliated cell populations that function to defend the airway against environmental stress [3], [7]–[9].

There are several lines of evidence that demonstrate controlling this differentiation process, in part, is through the canonical Wnt/β-catenin signaling pathway [10], [11]. The canonical Wnt pathway plays a central role in lung development and is critical for generation of the lung epithelium [10], [12]–[14]. Components of the canonical Wnt signaling pathway are expressed in embryonic and adult lung cell lines, as well as in the developing lung in a specific, spatio-temporal pattern [11], [15]–[17]. When canonical Wnt signaling is active, the pathway maintains cells in a low differentiation state [18]–[23]. However, when the canonical Wnt pathway is suppressed, undifferentiated progenitor cells are allowed to proceed toward differentiation [18]–[23]. Modulation of Wnt expression in embryonic and adult mouse lung suggests that the Wnt pathway is important for cell fate decisions and differentiation of lung cell type [24], [25]. The agonists of the canonical Wnt/β-catenin pathway include multiple Wnt proteins that function through frizzled receptors to up-regulate β-catenin, resulting in activation of the T cell factor (LEF1, TCF7, TCF7L1, TCF7L2) transcription factor family [26], [27]. The extracellular antagonists of Wnt signaling are the secreted fizzled-related (SFRP) and the Dickkopf (DKK) family of proteins [28], [29]. When the SFRPs and/or DKKs are up-regulated, the Wnt pathway is suppressed, allowing differentiation to proceed [28], [29].

Based on this background, we hypothesized that the canonical Wnt/β-catenin signaling pathway may be deranged in the airway epithelium when it is in a state of constant stress such as in cigarette smokers. To assess this hypothesis, we used microarrays to survey the mRNA levels of the canonical Wnt/β-catenin pathway in the small airway epithelium, the initial site of smoking-induced lung disease [30]. Pure populations of small airway epithelium were obtained by fiberoptic bronchoscopy of healthy nonsmokers, healthy smokers, and smokers with established COPD. The data demonstrate that most components of the Wnt/β-catenin pathway are expressed in the small airway epithelium. Interestingly, the downstream mediators β-catenin and the TCF transcription factor TCF7L1, as well as several downstream Wnt pathway target genes, are down-regulated in healthy smokers and smokers with COPD. Consistent with the down-regulation of Wnt/β-catenin pathway associated with smoking, the mRNA and protein levels of the Wnt inhibitor secreted frizzled-related protein 2 were greatly up-regulated in the small airway epithelium of healthy smokers and smokers with COPD, with expression primarily in ciliated cells. Finally, *in vitro* exposure of lung epithelium to cigarette smoke extract demonstrated similar results, with up-regulation of SFRP2 and down-regulation of the Wnt pathway. Together, these results show that smoking is associated with abnormal regulation of the Wnt pathway in the airway epithelium, an observation consistent with the disordered epithelial differentiation observed in smoking-related airway disorders.

## Methods

### Study Population

All individuals were evaluated at the Weill Cornell NIH Clinical and Translational Science Center and Department of Genetic Medicine Clinical Research Facility using protocols approved by the Weill Cornell Medical College Institutional Review Board. All subjects provided written consent before any study procedures were undertaken. Healthy nonsmokers and healthy smokers were characterized on the basis of clinical history and physical examination, routine blood screening tests, chest X-ray, electrocardiogram, urinalysis, and pulmonary function testing. Current smoking status was confirmed by history, venous carboxyhemoglobin levels, and urinalysis for nicotine levels and its derivative cotinine. Smokers with established COPD were defined according to Global Initiative for Chronic Obstructive Lung Disease criteria [31], [32].

### Collection of Small Airway Epithelium

Small airway epithelium was collected using flexible bronchoscopy as previously described [33], [34]. Smokers were asked not to smoke the evening prior to the procedure. A flexible bronchoscope (Pentax, EB-1530T3) was advanced to the desired bronchus after achieving mild sedation and anesthesia of vocal cords. Small airway samples were collected from 10^th^ to 12^th^ order bronchi using methods previously described. The airway epithelial cells were subsequently collected separately in 5 ml of LHC8 medium (GIBO, Grand Island, NY). An aliquot of this was used for cytology and differential cell count and the remainder was processed immediately for RNA extraction. Total cell counts were obtained using a hemocytometer while differential cell counts were determined on sedimented cells prepared by centrifugation (Cytospin 11, Shandon Instruments, Pittsburg, PA) and stained with DiffQuik (Baxter Healthcare, Miami, FL).

### RNA Extraction and Microarray Processing

Microarray analysis was performed using Affymetrix (Santa Clara, CA) microarray HG-U133 Plus 2.0 (54,675 probe sets) and associated protocols. Total RNA was extracted from epithelial cells using TRIzol (Invitrogen, Carlsbad, CA) followed by DNAnase (Qiagen, Valencia, CA) to remove residual DNA. An aliquot of each RNA sample was run on an Agilent Bioanalyzer (Agilent Technologies, Palo Alto, CA) to visualize and quantify the degree of RNA integrity. The concentration was determined using a NanoDrop ND-1000 spectrophotometer (NanoDrop Technologies, Wilmington, DE). Double-stranded complementary DNA was synthesized from 3 µg of total RNA using the GeneChip One-Cycle cDNA Synthesis Kit, followed by a cleanup step using GeneChip Sample Cleanup Module. Next, an *in vitro* transcription (IVT) reaction was performed with GeneChip IVT Labeling Kit after which further cleanup was carried out and quantification of the resulting biotin-labeled cRNA by spectrophotometry (all reagents from Affymetrix). Hybridizations to test chips and when permissible, to the microarrays, were conducted according to Affymetrix protocols. The Affymetrix GeneChip Fluidics Station 450 was used for processing the arrays with appropriate reagents/washes, prior to scanning with an Affymetrix GeneChip Scanner 3000 7G (http://affymetrix.com/support/technical/manual/expression_manual.affx).

Captured images were analyzed using Microarray Suite version 5.0 (MAS 5.0) algorithm (Affymetrix) as previously described. Samples used for analysis were required to satisfy quality control criteria including: (1) RNA Integrity Number (RIN) ≥7.0; (2) 3′/5′ ratio for GAPDH≤3; and (3) scaling factor ≤10.0 [35]. This data were normalized using GeneSpring version 7.3 software (Agilent Technologies, Palo Alto, CA) per array, by dividing the raw data by the 50^th^ percentile of all measurements. The data sets were assessed for expression of 56 Wnt signaling pathway genes and 55 Wnt target genes (based on the Wnt homepage, http://www.stanford.edu/~rnusse/wntwindow.html) using criteria of present (P call) of >20% of healthy nonsmokers.

### TaqMan RT-PCR Confirmation of Microarray Expression Levels

To confirm the microarray findings, TaqMan real-time RT-PCR was performed on RNA samples from the small airway samples of healthy nonsmokers, healthy smokers and smokers with COPD (n = 9 each, except for β-catenin, n = 11 each) that had been used for HG-U133 Plus 2.0 microarray analysis. First, cDNA was synthesized from 2 µg RNA in a 100 µl reaction volume, using the TaqMan Reverse Transcriptase Reaction Kit (Applied Biosystems), with random hexamers as primers. Dilutions of 1∶10 and 1∶100 were made from each sample and triplicate wells were run for each dilution. TaqMan PCR reactions were carried out using primers (Applied Biosystems, Foster City, CA), and 2 µl of cDNA was used in each 25 µl reaction volume. The endogenous control was 18S ribosomal RNA and relative expression levels were determined using the ΔΔCt method (Applied Biosystems). The rRNA probe was labeled with VIC and the probe for each gene of interest (β-catenin, TCF7L1, SOX9, MMP7) was labeled with FAM. The PCR reactions were run in an Applied Biosystems Sequence Detection System 7500.

### Western Analysis

Western analysis was used to quantitatively assess SFRP2 protein expression in small airway brushing samples from healthy nonsmokers, healthy smokers and smokers with COPD. Red blood cells were lysed (red blood cell lysis buffer, eBioscience, San Diego, CA), and proteins were extracted using RIPA buffer (Sigma-Aldrich, Saint Louis, MO) following the manufacture's instructions. Protein concentrations were assessed using a bicinchoninic acid (BCA) protein concentration kit (Pierce, Rockford, IL). Equal concentration of protein mixed with NuPAGE LDS Sample Buffer and NuPAGE Reducing Agent was heated at 70°C for 10 min. Then samples were loaded on NuPAGE 4 to12% Bis-Tris Gel (Invitrogen, Carlsbad, CA). Protein electrophoresis was carried out at 200 V, 1 hr, 23°C. Sample proteins were transferred (30 V, 1 hr, 4°C) to a 0.2 µm PVDF membrane (Invitrogen, Carlsbad, CA) in NuPAGE transfer buffer. The membranes were then blocked with 5% non-fat milk in Phosphate Buffered Saline with 0.1% tween-20 (PBST) for 1 hr. The membranes were incubated with primary rabbit polyclonal anti-SFRP2 antibodies (Santa Cruz Biotechnology, Santa Cruz, CA) at 1∶200 dilution 4°C overnight. Detection was performed using secondary horseradish peroxidase-labeled goat anti-rabbit antibody (1∶2,000 dilution, Santa Cruz Biotechnology) and then enhanced chemiluminescent reagent system (GE Healthcare, Pittsburgh, PA). To assess the Western analysis quantitatively, the film was digitally imaged, maintaining exposure within the linear range of detection. The contrast was inverted, the pixel intensity of each band determined, and the background pixel intensity for a negative area of the film of identical size subtracted using MetaMorph image analysis software (Universal Imaging, Downingtown, PA). The membrane was subsequently stripped and reincubated with monoclonal anti-β-actin antibody (Sigma-Aldrich, Saint Louis, MO) as a control for equal protein concentration.

### Localization of SFRP2 in the Airway Epithelium

To determine the distribution of SFRP2 expression in small airway epithelial cells, cytospin preparation of bronchial brushes obtained from the small airway epithelium of healthy nonsmoker, healthy smokers and smokers with COPD were assessed by SFRP2-specific immunofluorescence. Sections were fixed in 4% paraformaldehyde for 15 min, and washed twice in PBS. Samples were blocked in normal goat serum to reduce background staining and then incubated with the rabbit anti-human SFRP2 primary antibody (Atlas Antibodies, Stockholm, Sweden) diluted 1∶250 and rabbit IgG (Jackson Immunoresearch, West Grove, PA) as the isotype control at 4°C overnight. Goat anti-rabbit Cy3 conjugated AffiniPure IgG (Jackson ImmunoResearch) at 1∶50 dilution was used as a secondary antibody for SFRP2. Nuclei were counter stained with 4′, 6-diamidino-2-phenylindole (DAPI, 1∶2,000 dilution; Invitrogen, Carlsbad, CA). Images were captured using an Olympus IX 70 fluorescence microscope with 60-fold magnification. Images were analyzed using MetaMorph software (Universal Imaging Corporation, Downingtown, PA).

As a further assessment at the morphologic level, bronchial biopsies from large airway of healthy nonsmokers, healthy smokers and smokers with COPD were assessed by immunofluorescence for SFRP2. Sections were deparaffinized and rehydrated through a series of xylenes and alcohol. Further staining procedures were as described above.

Colocalizations of SFRP2 with a ciliated cell-specific marker β-tubulin IV, secretory cell-specific marker mucin 5AC and neuroendocrine-specific marker chromogranin A were also performed with cytospin preparations and biopsies. The following antibodies were used: for β-tubulin IV, mouse monoclonal anti-human β-tubulin IV (1∶2000; Biogenex, San Ramon, CA) and mouse IgG as the isotype control (Sigma, St Louis, MO); for mucin 5AC, mouse monoclonal (CLH2) anti-human mucin 5AC (1∶50; Vector Laboratories, Burlingame, CA) and mouse IgG as the isotype control (Sigma, St Louis, MO); for chromogranin A, mouse monoclonal (LK2H10+PHE5) anti-human chromogranin (1∶500 dilution; Thermo Scientific, Waltham, MA) and mouse IgG as the isotype control (Sigma, St Louis, MO). Following incubation with the primary antibodies, goat anti-rabbit Cy5 (Jackson ImmunoResearch) was used as a secondary antibody for SFPR2 and goat anti-mouse Cy3 (Jackson ImmunoResearch) was used as a secondary antibody for β-tubulin IV, mucin 5AC and chromogranin A. Further staining procedures were as described above.

### Down-regulation of Wnt Pathway Target Genes in Human Small Airway Epithelial Cells Exposed to Cigarette Smoke Extract *In Vitro*

Aqueous cigarette cell extract (CSE) was generated from the combustion of 1 cigarette (Marlboro Red) bubbled through 12.5 ml of culture medium [36]. This medium, defined as “100% CSE,” was adjusted to pH 7.4 and filtered through a 0.22 µm filter. Different concentrations of CSE diluted with the culture medium were employed, ranging from 0.1 to 20%. The human airway epithelial cell line 16HBE [37] was exposed to freshly prepared CSE for 72 hr. Cell viability was assessed by 3-(4,5-dimethylthiazol-2-yl)-2,5-diphenyltetrazolium bromide (MTT) assay (Roche Applied Science) [38]. Viability was expressed as percentage of the values (corresponding to 100%) of untreated cells. SFRP2, MMP7, SOX9 gene expression was assessed with TaqMan real-time PCR.

### Wnt Reporter Assay

HEK293 cells were transiently transfected with the reporter construct Topflash or Fopflash (kindly provided by R. Moon, University of Washington, Seattle) using Lipofectamine LTX (Invitrogen, Carlsbad, CA) [39]. The Topflash construct contains 8 Tcf/Lef binding sites upstream of a minimal TA viral promoter and the firefly luciferase cDNA. The Fopflash construct is identical except that it contains mutated copies of TCf/Lef binding sites and is used as a control for measuring nonspecific activation of the reporter construct. Expression of Renilla luciferase provides an internal control value to which expression of the experimental firefly luciferase reporter gene will be normalized. The experiment was divided into 6 groups: Topflash plus CMV-renilla; Fopflash plus CMV-renilla; Topflash plus CMV-renilla plus WNT1; Fopflash plus CMV-renilla plus WNT1; Topflash plus CMV-renilla plus WNT1 plus SFRP2; Fopflash plus CMV-renilla plus WNT1 plus SFRP2. After 48 hr, luciferase activities were determined using the Dual Luciferase Assay System (Promega) on a tube luminometer (Berthold detection systems).

### Statistical Analysis

HG-U133 Plus 2.0 microarrays were analyzed using GeneSpring software. Average expression values were calculated from normalized expression levels for healthy nonsmokers, healthy smokers, and smokers with COPD. p values were obtained using Benjamini-Hochberg correction to limit the false positive rate. Statistical comparisons between continuous variables were calculated using an unpaired, two-tailed t-test with unequal variance. Statistical comparisons for categorical data were achieved using Chi-squared test. A p value<0.05 was considered significant. p values for TaqMan data were calculated using two-tailed Student's t-test.

### Web Deposition of Data

All MIAME-compliant microarray data have been deposited in the Gene Expression Omnibus (GEO) site (http://www.ncbi.nlm.nih.gov/geo), which is curated by the National Center for Bioinformatics. Accession number for small airways HG-U133 Plus 2.0 is GSE19407.

## Results

### Study Population

Small airway samples from a total of 127 individuals, including 47 healthy nonsmokers, 58 healthy smokers and 22 smokers with established COPD were analyzed with microarray HG-U133 Plus 2.0 (Table 1). There were no differences with respect to gender among the groups (p>0.3), but the COPD smokers were older than the other groups (p<0.05). There were no differences among groups with regard to ancestral background (p>0.3). All individuals were HIV negative. Smokers had urine nicotine and cotinine and venous blood carboxyhemoglobin levels confirming their current smoking status.

**Table 1:** Demographics of the Study Population and Biologic Samples^1^.

Parameter	Healthy nonsmokers	Healthy smokers	COPD smokers^2^
n	47	58	22
Sex (male/female)	33/14	38/20	18/4
Age (yr)	41.9±11.4	42.9±7.2	51.5±8.5
Race (B/W/O)^3^	23/18/6	35/14/9	8/9/5
Smoking history (pack-yr)	-	27.5±16.6	40.9±28.2
Urine nicotine (ng/ml)	-	1298±1676	1114±1132
Urine cotinine (ng/ml)	-	1246±965	1357±598
Blood carboxyhemoglobin (%)	0.4±0.7	1.8±1.9	3.0±2.0
Pulmonary function parameters^4^			
FVC	107±14	109±13	97±20
FEV1	106±15	107±14	74±21
FEV1/FVC	82±6	80±5	61±9
TLC	101±13	100±12	102±22
DLCO	99±15	94±11	75±19
Gold stage (I/II/III)^2^	-	-	9/11/2
Medication use			
β-agonist	-	-	7
Anticholinergic	-	-	2
Inhaled corticosteroid	-	-	3
Epithelial cells^5^			
Number recovered×10^6^	6.4±2.9	7.2±3.0	6.7±3.2
% epithelial cells^6^	99.3±1.1	99.1±1.3	98.9±1.4
% inflammatory cells	0.7±1.1	0.8±1.3	1.1±1.4
Differential cell count			
Ciliated (%)	74±7.4	65.7±12.4	63.5±10.9
Secretory (%)	6.7±3.5	9.1±4.5	11.9±5.6
Basal (%)	11.1±5.3	12.7±6.6	11.9±6.3
Undifferentiated (%)	7.3±3.2	11.9±6.6	11.6±3.7

1 Data are presented as mean ± standard deviation.

2 Smokers with “established COPD” defined by the GOLD criteria [31], [32]; the COPD smoker group included: GOLD I n = 9, GOLD II n = 11, and GOLD III n = 2.

3 B = black, W = white, O = other.

4 Pulmonary function testing parameters are given as % of predicted value with the exception of FEV1/FVC, which is reported as % observed; FVC - forced vital capacity, FEV1 - forced expiratory volume in 1 sec, TLC - total lung capacity, DLCO - diffusing capacity. For individuals with COPD, FVC, FEV1, and FEV1/FVC are post-bronchodilator values.

5 Small airway epithelium.

6 As a % of small airway epithelium recovered.

### Sampling of Airway Epithelium

Airway epithelial cells were obtained by fiberoptic bronchoscopy and brushing of small (10^th^ to 12^th^ order) airways. The number of cells recovered averaged from 2.1 to 21×10^6^, with an average of >96% epithelium (Table 1). The various categories of airway epithelial cells were, as expected, from the small airway epithelium [33], [34].

### Expression of Wnt Signaling Pathway Genes in Small Epithelium of Healthy Nonsmokers, Healthy Smokers and Smokers with COPD

**Figure 1:**
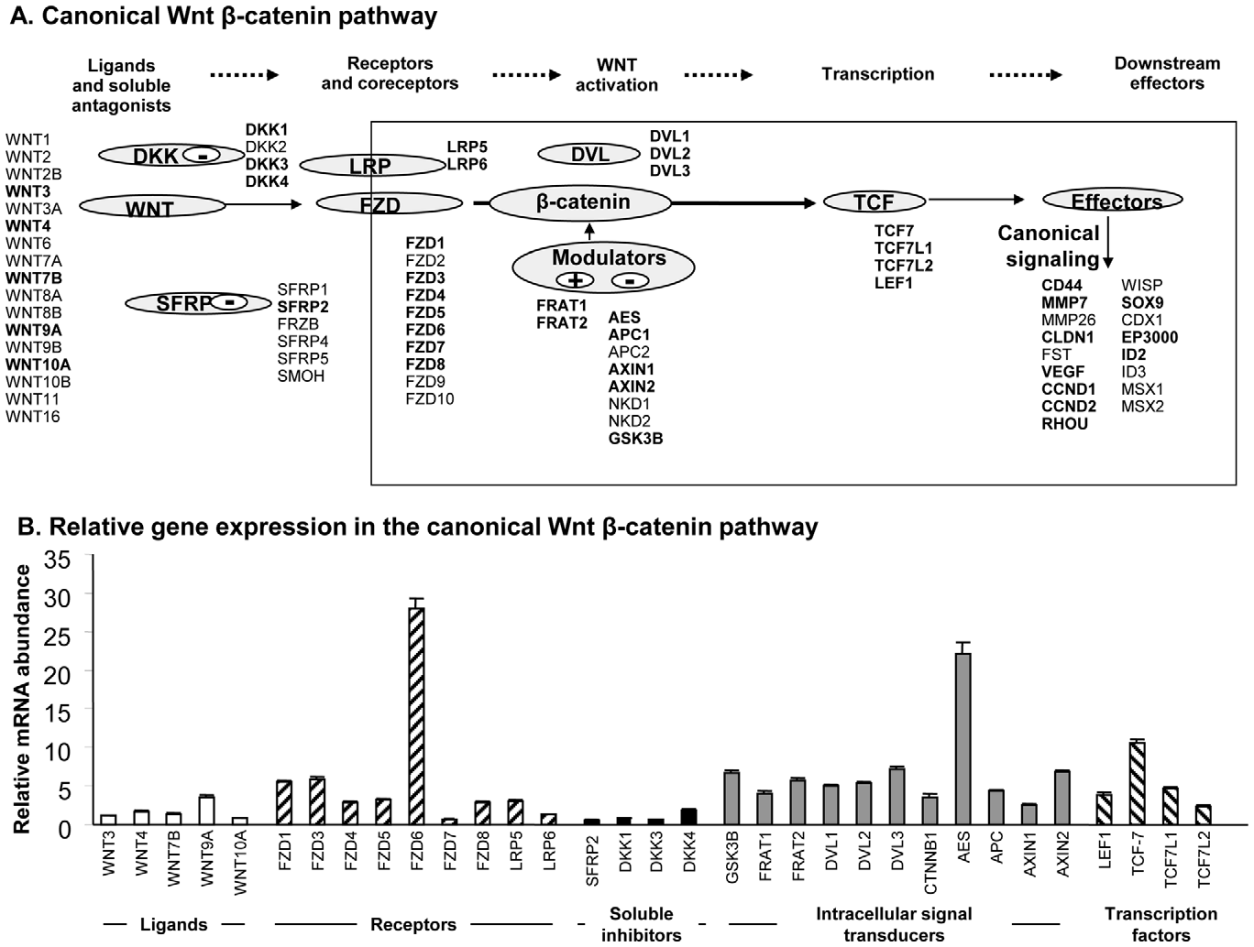
The Wnt pathway expression in the small airway epithelium of human healthy nonsmokers. **A**. Schematic of the pathway, showing which (in bold) ligands, soluble antagonists, receptors, co-receptors, intracellular activators, transcription factors and downstream effectors are expressed. **B**. Gene expression in canonical Wnt β-catenin pathway in healthy nonsmokers in small airway epithelium. Small airway epithelium from 47 normal nonsmokers were analyzed using the HG-U133 Plus 2.0 array and expression level normalized by chip only is plotted. Error bars represent the standard error.

The genome-wide microarrays were used to analyze the gene expression profiles of canonical Wnt signaling components at the mRNA level in the small airway epithelium of healthy nonsmokers, healthy smokers, and smokers with established COPD. In the small airway epithelium of healthy nonsmokers, 33 of 56 (60%) genes in the Wnt pathway were expressed (Table S1). These expressed genes included Wnt extracellular ligands (WNT3, 4, 7B, 9A, 10A), frizzled receptors (FZD1, 3, 4, 5, 6, 7, 8), co-receptors (LRP5, LRP6), extracellular inhibitors (SFRP2, DKK1, DKK3, DKK4), intracellular mediators (β-catenin, GSK-3B, DVL1, 2, 3, AES, APC1, AXIN1, AXIN2, FRAT1, FRAT2), nuclear transcription factors (TCF7, TCF7L1, TCF7L2, LEF1). For Wnt target genes, 27 out of 55 genes are present in small airway epithelium, including MMP7, CLDN1, VEGFA, CCND1, SOX9, ID2 (Figure 1A, Table S1).

In healthy nonsmokers the relative expression of WNT9A was the most highly expressed Wnt ligand, with average expression approximately 3.3-fold greater than WNT3, and 4.7-fold greater than WNT10A (both p<0.0001). For receptors, the most highly expressed receptor was FZD6, which was expressed 44.4-fold higher than FZD7 (p<0.0001), the lowest expressed receptor. For transcription factors in the Wnt pathway, TCF7 was the downstream gene most highly expressed in the small airway samples, 2.3-fold more highly expressed than TCF7L1, 4.4-fold more than TCF7L2, and 2.7-fold more than LEF1 (p<0.0001, all comparisons).

**Figure 2:**
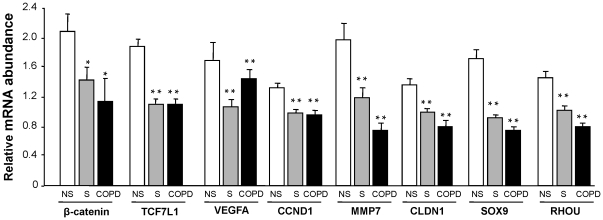
Comparison of the relative expression of the Wnt pathway downstream and target genes in healthy nonsmokers (n = 47), healthy smokers (n = 58), and smokers with COPD (n = 22). Analysis was carried out with HG-U133 plus 2.0 microarrays normalized by chip and gene. Each bar represents mean expression with standard error; * p<0.05 compared to healthy nonsmokers, * * p<0.01 compared to healthy nonsmokers.

Compared to healthy nonsmokers, the Wnt intracellular modulators, transcription factors and target genes were down-regulated in healthy smokers and smokers with established COPD. β-catenin, a central molecule in the Wnt signaling pathway, was down-regulated 1.5-fold in healthy smokers and 1.8-fold in smokers with COPD (both comparisons, p<0.05, Figure 2, Table 2). Consistent with this observation, TCF7L1, a transcription factor in Wnt pathway, was down-regulated 1.7-fold in smokers and 1.7-fold in smokers with established COPD (p<0.01, Figure 2, Table 2). Wnt target genes, including MMP7, VEGFA, CLDN1, CCND1, SOX9, RHOU, were also down-regulated in healthy smokers and smokers with COPD compared to healthy nonsmokers (p<0.05 for VEGFA and p<0.01 for the other genes; Figure 2, Table 3). The network of the WNT/β-catenin genes in healthy smokers and smokers with COPD compared to healthy nonsmokers were analyzed with Ingenuity Pathway Analysis (Ingenuity Systems, http://www.ingenuity.com; Figure S1).

**Table 2:** Small Airway Epithelium Wnt Pathway Genes with Suppressed Expression in Healthy Smokers and COPD Smokers Compared to Healthy Nonsmokers^1^.

Category	Gene symbol	Gene title	Probeset ID	P call (%)			Smoker/nonsmoker		COPD/nonsmoker	
				Nonsmoker	Smoker	COPD smoker	Fold-change	p value	Fold-change	p value
Receptors	FZD1	frizzled homolog 1 (Drosophila)	204451_at	98	93	100	−1.17	<0.01	−1.13	NS
	FZD4	frizzled homolog 4 (Drosophila)	218665_at	100	97	100	−1.15	<0.05	−1.23	<0.001
	FZD6	frizzled homolog 6 (Drosophila)	203987_at	100	100	100	−1.14	<0.05	−1.16	NS
	FZD8	frizzled homolog 8 (Drosophila)	224325_at	100	95	91	−1.54	<0.0001	−1.41	<0.01
Intracellular modulators	FRAT1	frequently rearranged in advanced T-cell lymphomas	219889_at	100	100	100	−1.22	<0.01	−1.14	NS
	FRAT2	frequently rearranged in advanced T-cell lymphomas 2	209864_at	100	100	100	−1.34	<0.0001	−1.22	<0.05
	DVL1	dishevelled, dsh homolog 1 (Drosophila)	203230_at	100	100	100	−1.17	<0.05	−1.04	NS
	DVL3	dishevelled, dsh homolog 3 (Drosophila)	201908_at	100	100	100	−1.24	<0.001	−1.18	<0.05
	β-catenin	catenin (cadherin-associated protein), beta 1, 88 kDa	1554411_at	96	88	64	−1.46	<0.05	−1.84	<0.05
	AXIN2	axin 2	222696_at	100	97	100	−1.16	<0.05	−1.22	<0.01
Transcription factors	TCF7L1	transcription factor 7-like 1 (T-cell specific, HMG-box)	221016_s_at	100	100	100	−1.71	<0.0001	−1.72	<0.0001

1 NS, not significant.

**Table 3:** Small Airway Epithelium Wnt Pathway Target Genes with Suppressed Expression in Healthy Smokers and COPD Smokers Compared to Healthy Nonsmokers^1^.

Gene symbol	Gene title	Probe set ID	P call (%)			Smoker/nonsmoker		COPD/nonsmoker	
			Non-smoker	Smoker	COPD smoker	Fold-change	p value	Fold-change	p value
SOX9	SRY (sex determining region Y)-box 9	202936_s_at	100	100	100	−1.88	<0.0001	−2.29	<0.0001
RHOU	ras homolog gene family, member U	223168_at	100	100	100	−1.60	<0.0001	−1.88	<0.0001
RUNX2	runt-related transcription factor 2	232231_at	100	100	100	−1.51	<0.0001	−1.32	<0.0
JAG1	jagged 1	209099_x_at	100	100	100	−1.33	<0.0001	−1.32	<0.01
CCND1	cyclin D1	208712_at	100	100	100	−1.34	<0.0001	−1.39	<0.001
CLDN1	claudin 1	218182_s_at	98	97	86	−1.37	<0.0001	−1.70	<0.0001
CDH1	cadherin 1, type 1, E-cadherin (epithelial)	201131_s_at	100	100	100	−1.25	<0.001	−1.14	NS
AXIN2	axin 2	222696_at	100	97	100	−1.16	<0.05	−1.22	<0.01
MMP7	matrix metallopeptidase 7 (matrilysin, uterine)	204259_at	47	14	9	−1.66	<0.01	−2.62	<0.0001
ID2	inhibitor of DNA binding 2, dominant negative helix-loop-helix protein	201565_s_at	100	100	100	−1.16	<0.05	−1.12	NS
VEGFA	vascular endothelial growth factor A	210512_s_at	100	100	100	−1.58	<0.05	−1.17	NS
SOX2	SRY (sex determining region Y)-box 2	228038_at	100	100	100	−1.12	<0.05	−1.07	NS

1 NS, not significant.

Modulation of the Wnt signaling pathway in the small airway epithelium of healthy smokers and smokers with COPD compared to healthy nonsmokers was verified using TaqMan PCR for selected central genes in the pathway (Figure S2). Consistent with microarray data, β-catenin was down-regulated 1.7-fold in smokers compared to nonsmokers, and 1.9-fold in smokers with COPD compared with healthy nonsmokers (p<0.05). For transcription factors, TCF7L1 was confirmed to be down-regulated in healthy smokers and smokers with COPD compared to healthy nonsmokers (p<0.05). Wnt signaling pathway target genes were also confirmed to be down-regulated. MMP7 expression decreased 7.5-fold in healthy smokers and 12.9-fold in smokers with COPD compared to healthy nonsmokers (p<0.05). SOX9, another Wnt target gene, was also confirmed to be down-regulated in the small airway epithelium of healthy smokers and smokers with COPD compared to healthy nonsmokers (3.9-fold for smokers *vs* nonsmokers and 3.2-fold for smokers with COPD *vs* nonsmokers, both p<0.01).

### Expression of SFRP2 in the Small Airway Epithelium of Healthy Nonsmokers, Healthy Smokers and Smoker with COPD

**Figure 3:**
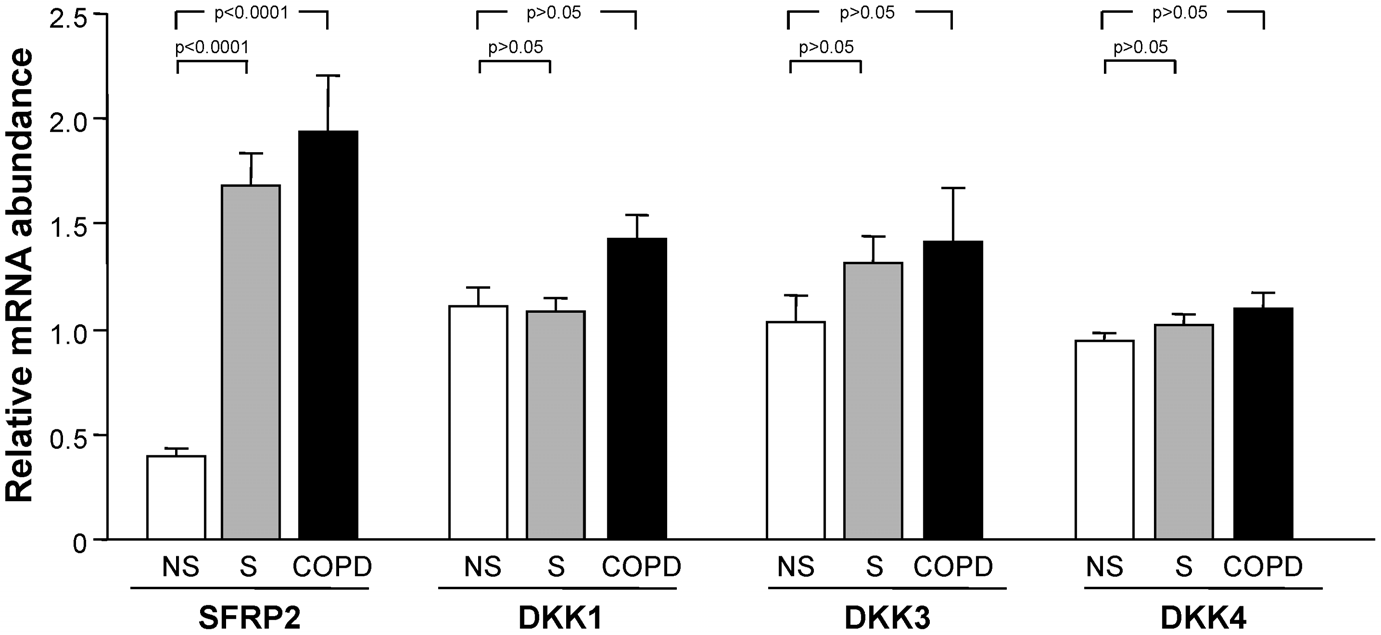
Comparison of the relative expression of the Wnt pathway inhibitors in healthy nonsmokers (n = 47), healthy smokers (n = 58), and smokers with COPD (n = 22). Analysis was carried out with HG-U133 plus 2.0 microarrays. Each bar represents mean expression with standard error; p values are represented in brackets above the bars.

The *in vivo* gene expression data showed that Wnt signaling pathway was down-regulated in healthy smokers and smokers with COPD compared to healthy nonsmokers in the small airway epithelial cells. As a possible mechanism to explain this down-regulation, we hypothesized that there may be smoking-induced up-regulation of the SFRP and/or DKK family of extracellular Wnt pathway inhibitors. To assess this, we analyzed Wnt inhibitors expressed in human small airway epithelium including, SFRP2, DKK1, DKK3 and DKK4. Interestingly, among these 4 inhibitors, SFRP2 showed significant gene expression change between healthy smokers and healthy nonsmokers, with SFRP2 up-regulation 4.3-fold in healthy smokers and 4.9-fold in smokers with COPD compared with healthy nonsmokers (p<0.0001, Figure 3).

**Figure 4:**
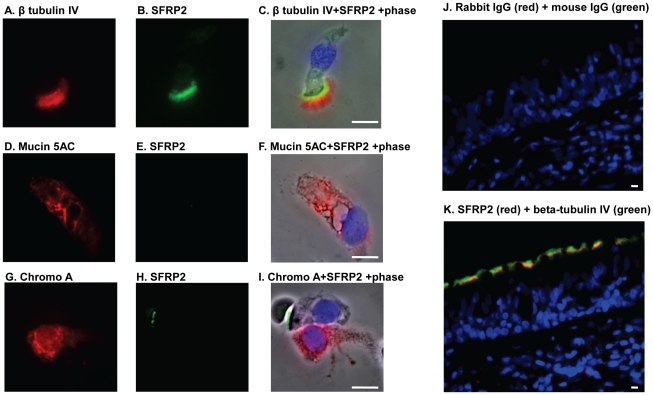
Co-localization of SFRP2 and ciliated cell specific marker β-tubulin IV. For Figures 4**A–I**, Cytospin preparations of airway epithelium from a healthy smoker were stained with antibodies against SFRP2 (green), β-tubulin IV (red), Mucin 5AC (red) and chromogranin A (red). **A–C**. Colocalization of SFRP2 and β-tubulin IV; **D–F**. Colocalization of SFRP2 and mucin 5AC; **G–I**. Colocalization of SFRP2 and chromogranin A. For Figure 4**J–K**, biopsies from a healthy smoker were stained with antibodies against SFRP2 (red) β- tubulin IV (green). **J**. IgG controls of SFRP2 and β-tubulin IV; and **K**. SFRP2 and β-tubulin IV co-localization.

**Figure 5:**
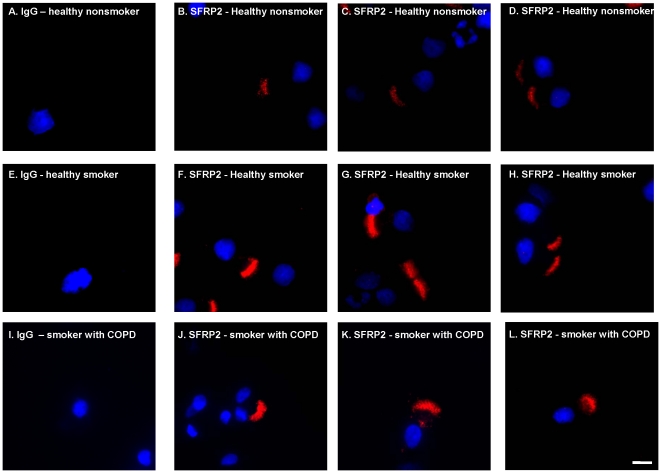
Immunofluorescent assessment of SFRP2 expression in cytospin preparations of brushed small airway epithelium. Small airway epithelial cell cytopreparations of healthy nonsmokers, healthy smokers and smokers with COPD were stained with anti-SFRP2 followed by a Cy3 conjugated secondary antibody (shown in red). Nuclei were stained with DAPI (shown in blue) **A–D**. Healthy nonsmokers. **A**. IgG control; **B–D**. Examples of anti-SFRP2. **E–H**. Healthy smokers. **E**. IgG control; **F–H**. Examples of anti-SFRP2. **I–L**. Smokers with COPD; **I**. IgG Control. **J–L**. Examples of anti-SFRP2. Bar = 10 µm.

**Figure 6:**
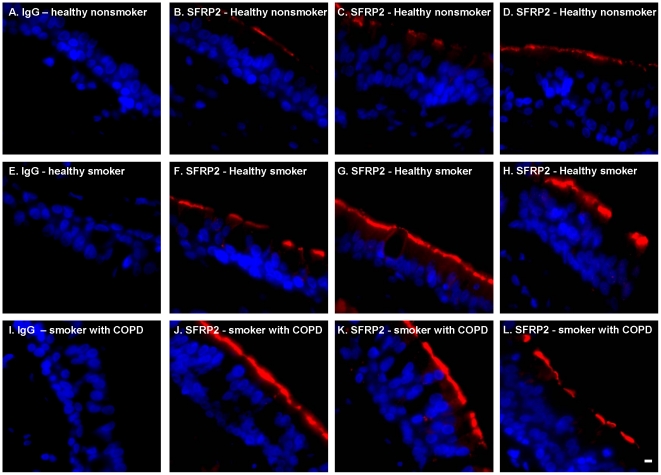
Immunofluorescent assessment of SFRP2 expression in the endobronchial biopsies from large airway epithelium. Endobronchial biopsies from large airway of healthy nonsmoker, healthy smokers and smokers with COPD were stained with anti-SFRP2 followed by a Cy3 conjugated secondary antibody (shown in red). Nuclei were stained with DAPI (shown in blue). **A–D**. Healthy nonsmokers. **A**. IgG control; **B–D**. Examples of anti-SFRP2. **E–H**. Healthy smokers. **E**. IgG control; **F–H**. Examples of anti-SFRP2. **I–L**. Smokers with COPD. **I**. IgG Control, **J–L**. Examples of anti-SFRP2. Bar = 10 µm.

To confirm the results obtained from microarrays, anti-SFRP2 immunofluorescence and Western analysis were utilized. First, anti-SFRP2 immunofluorescence was carried out on cytospin preparations of small airway epithelial cells obtained from healthy nonsmokers, healthy smokers and smokers with COPD. In all samples, SFRP2 protein expression was detected specifically in ciliated cells; other cell types, including secretory cells, undifferentiated cells and basal cells, did not show obvious staining (Figure 4, Figure 5). Consistent with the up-regulation of SFRP2 mRNA, qualitative immunofluorescence assessment suggested that SFRP2 protein was up-regulated in the healthy smokers and COPD smokers. Second, as further confirmation, SFRP2 expression was also observed to be up-regulated in ciliated cells of large airway biopsies (Figure 6).

**Figure 7:**
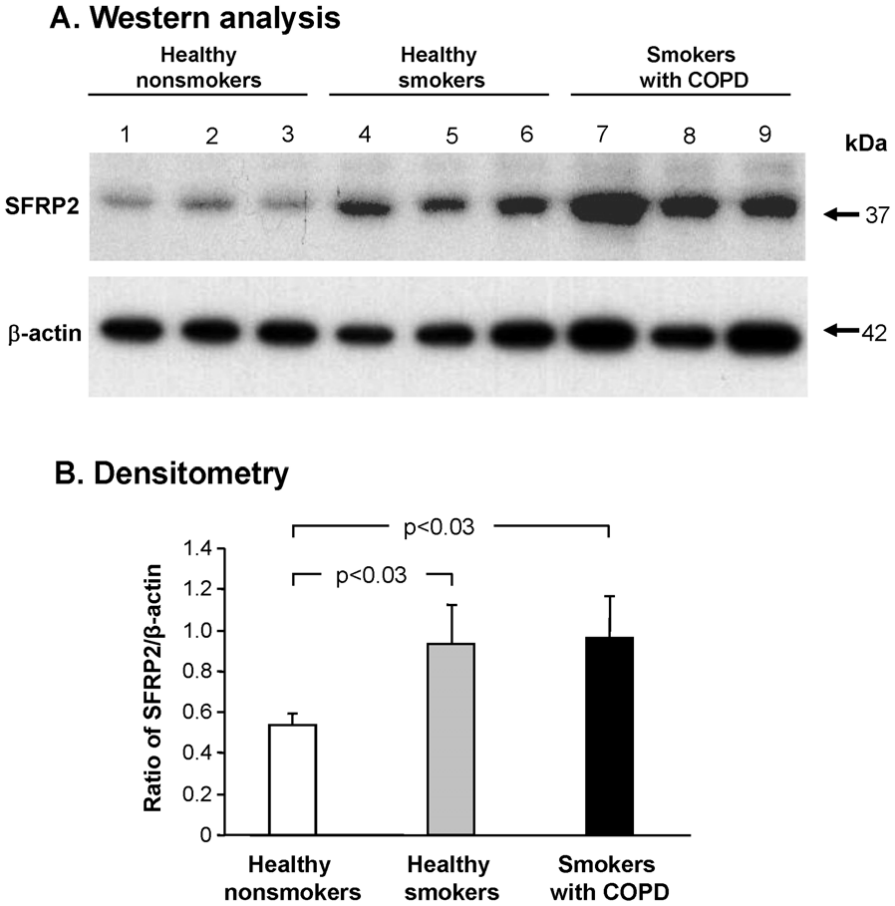
Western analysis of SFRP2 protein expression in small airway epithelial cells. **A**. Proteins were extracted from small airway epithelial cells of 3 healthy nonsmokers, 3 healthy smokers and 3 smokers with COPD. Shown is SFRP2 protein expression in healthy nonsmokers (lanes 1–3), healthy smokers (lanes 4–6) and smokers with COPD (lanes 7–9). Lower panel - same membrane probed with anti β-actin antibody, a control for protein loading. **B**. Quantification by densitometry of SFRP2 to β-actin. The ratio for SFRP2 to β-actin based on panel A is represented on the ordinate for the nonsmoker, smoker and smoker with COPD. Error bars represent the standard error.

To quantify this observation, Western analysis of SFRP2 was carried out on small airway epithelial cell samples from healthy nonsmokers, healthy smokers and smokers with COPD. This analysis demonstrated increased SFRP2 protein expression in healthy smokers and smokers with established COPD compared to healthy nonsmokers in small airway epithelial cells (Figure 7, p<0.05).

### Down-regulation of the Wnt Signaling Pathway in Human Airway Epithelial Cells Exposed to Cigarette Smoke Extract *In Vitro*

**Figure 8:**
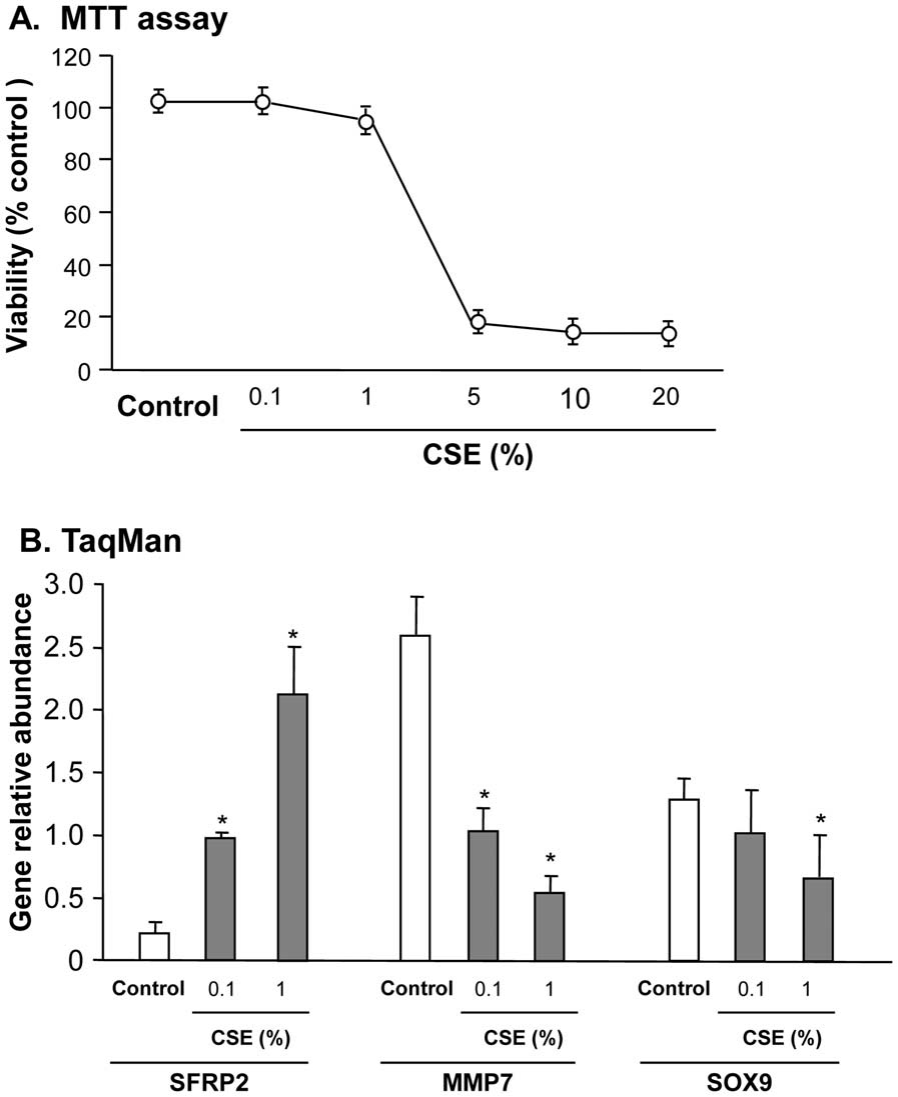
*In vitro* cigarette smoke extract (CES) treatment of human 16HBE airway epithelial cells. After 16HBE cells were exposed to different concentrations of CSE for 3 days, cell viability (MTT) and gene expression levels of Wnt signaling pathway inhibitor SFRP2 and target genes (MMP7, SOX9) were quantified using TaqMan real-time PCR. **A**. MTT assessment. **B**. Effect of CSE on gene expression of SFRP2, MMP7, SOX9 in 16HBE cells using 0.1 and 1% CSE concentrations that do not affect viability. SFRP2 gene expression was up-regulated when treated with both 0.1 and 1% CSE (4.6-fold and 10.1-fold, p<0.01 and p<0.001). Error bars represent the standard deviation. *p<0.05 compared to no CSE controls. For TaqMan real-time PCR, the average value of 0.1% CSE treatment group for each gene is determined as the calibrator.

To assess whether the exposure of human airway epithelial cells to CSE would affect Wnt pathway gene expression, cultures of the human airway epithelial cell line 16HBE were exposed to freshly made CSE for 72 hr. Cell viability was analyzed with MTT assay and SFRP2, MMP7 and SOX9 gene expression was analyzed using TaqMan real-time PCR. Decreased cell viability compared to control cells was observed with concentrations of 5, 10 and 20% CSE. Cell viability was 100% with 0.1% CSE and 92% with 1% CSE medium compared with controls (Figure 8A). Based on this data, we chose 0.1% and 1% CSE for the CES *in vitro* exposure studies. Compared with controls, SFRP2 gene expression increased in a dose-dependent pattern, with 4.6-fold in 0.1% CSE group (p<0.01) and 10.1-fold in 1% CSE groups (p<0.001) compared to no CSE controls (Figure 7B). The Wnt target gene MMP7 decreased 2.3-fold (p<0.05) and 4.0-fold (p<0.01) respectively in 0.1% and 1% CSE groups compared to the control. SOX9, another Wnt target gene, was not significant in 0.1% CSE group, but significantly decreased in 1% CSE group (1.8-fold, p<0.05) compared to the control (Figure 8B).

### Wnt Reporter Assay

To demonstrate SFRP2-dependent suppression of Wnt signaling, HEK293 cells were transiently transfected with the reporter constructs. When luciferase expression was driven by a WNT1-dependent promoter, the co-transfection of SFRP2 in addition to the WNT1 plasmid decreased luciferase activity in HEK293 cells by two fold (p<0.0001, Figure S3).

## Discussion

In healthy individuals, as airway epithelial cells are injured or reach senescence, there is ongoing cell regeneration, with basal cell proliferation and subsequent differentiation to ciliated and secretory cells that comprise the healthy pseudostratified epithelial barrier that lines the airways [1]–[3], [5]. In cigarette smokers, the toxic components of smoke and the associated local persistent inflammatory host response to smoking place the epithelium under the constant stress of oxidants, apoptotic signals, and other mechanisms of injury [40]–[43]. In response, the airway epithelium regenerates at a faster rate, attempting to maintain its healthy pseudostratified character [40], [44]. To assess the role of the Wnt pathway in the context of the stress of cigarette smoking, whole-genome microarray was utilized to analyze expression of the Wnt pathway genes. The data demonstrate that the majority of genes involved in the Wnt signaling pathway, representing all functional categories in the pathway, are expressed in the normal adult human small airway epithelium of healthy nonsmokers, consistent with studies showing the Wnt pathway can regulate human lung morphogenesis [11], [24], [25]. With the stress of smoking, the Wnt pathway intracellular modulators β-catenin and transcription factor TCF7L1, as well as Wnt target genes are down-regulated, suggesting the Wnt pathway is suppressed. Assessment of the known extracellular inhibitors of the Wnt pathway revealed that smoking is associated with up-regulation of SFRP2. The up-regulated SFRP2 expression occurred in the ciliated cell population, suggesting that the differentiated cells play a role in modulating the “on” and “off” status of the Wnt pathway. Finally, *in vitro* exposure of human airway epithelial cells to cigarette smoke extract recapitulated the *in vivo* observations, with increased SFRP2 gene expression and down-regulation of Wnt target gene expression, demonstrating that cigarette smoke can directly down-regulate Wnt signaling pathway in the airway epithelium.

### Wnt Signaling Pathway and Lung Development

Wnt pathway signaling is an important regulator of epithelial and mesenchymal cell biology [26], [27]. To date, there are at least 19 Wnt ligands and 10 Fz receptor proteins identified, with redundancy and multiplicity of the receptor interactions and the downstream signaling [26], [27]. Of the various Wnt mutant phenotypes in mice, loss of the ligands WNT7B and WNT5A results in an abnormal pulmonary phenotype [15], [24]. Wnt7B ^lacZ−/−^ mice exhibit lung hypoplasia and respiratory failure [24], and Wnt7B^lacZ−/−^ embryos and newborn mice exhibit severe defects in the smooth muscle component of the major pulmonary vessels, suggesting that WNT7B signaling is required for proper lung mesenchymal growth and vascular development [24]. Mice carrying a targeted disruption of the WNT5A locus show abnormalities in distal lung morphogenesis, as manifested by truncation of the trachea and overexpansion of the distal respiratory airways [15]. The overall architecture of these mutant lungs is characterized by overexpansion of the distal airways and inhibition of lung maturation, with a persistence of thickened intravascular interstitium [15]. Activation of β-catenin in mice causes ectopic differentiation of alveolar type II-like cells in conducting airways, goblet cell hyperplasia, and air space enlargement, suggesting a critical role for the Wnt/β-catenin signal transduction pathway in the differentiation of the respiratory epithelium in the postnatal lung [12].

### Wnt Signaling Pathway and Lung Disease

Loss of regulation of Wnt signaling pathways has been linked to the pathogenesis of asthma, fibrotic lung disease, nitrogen-induced pulmonary hypoplasia and lung cancer [45]–[50]. Konigshoff and colleagues [48], [50] confirmed the up-regulation of WNT1, WNT7B and WNT10B, FZD2, FZD3, β-catenin, and LEF1 expression in the lungs of individuals with idiopathic pulmonary fibrosis compared to transplant donor lung. *In vitro* functional studies have indicated that Wnt ligands induce lung epithelial cell proliferation and (myo) fibroblast activation and collagen synthesis [48]. Increased expression of Wnt ligands (WNT1, 2, 7A) along with decreased expression of Wnt inhibitors (DKK3 and SFRP3) was observed in non-small cell lung cancer [47], [49], [51]–[53].

### Function of Wnt Signaling Pathway in Adult Human Airway Epithelium

In a previous study from our laboratory, the large airway epithelium of healthy individuals was denuded by bronchoscopy and brushing, and genome-wide microarrays were used to study the gene expression pattern in the same site at 0, 7 and 14 days after injury [54]. Histologically, the injured area was completely covered by a partially redifferentiated epithelial layer after 7 days, and at this time point, a Wnt pathway inhibitor DKK1 was found to be greatly up-regulated, suggesting that the Wnt/β-catenin pathway is inhibited at this stage of airway epithelial repair [54].

In the present study, we found that the Wnt signaling pathway was also down-regulated in adult human airway epithelium under the stress of chronic smoking. Interestingly, using gene expression profiling with air/whole mainstream cigarette smoke to treat a three-dimensional air-liquid interface model of tracheobronchial epithelium that were grown from primary human lung epithelial cells, Maunders et al [55] showed that the Wnt signaling pathway was down-regulated at 1, 6, and 24 hr post-exposure. For example, at 1 hr post-treatment, 9 Wnt signaling pathway genes and 12 Wnt target genes, including AXIN2, CCND1, JAG1, RUNX2, were down-regulated, with no up-regulation of Wnt signaling pathway genes or Wnt target genes. This supports our *in vivo* data that smoking repressed the Wnt signaling pathway. We also showed that *in vitro* smokers have significant up-regulation of another Wnt pathway inhibitor, SFRP2, an extracellular mediator of the largest family of Wnt inhibitors. SFRP2 functions as a modulator of Wnt signaling through direct interaction with Wnt ligands, with consequent regulation of cell growth [28], [56]–[64]. Inactivation of SFRP2 results in subtle limb defects in mice with mesomelic shortening and consistent shortening of all autopodal elements clinically manifested as brachydactyly [65]. SFRP2 has been shown to repress canonical Wnt signaling activated by WNT1, WNT3A, WNT4, WNT7A, and WNT9A *in vitro* [65]–[67]. Interestingly, SFRP1, another member of the SFRP family, is up-regulated in the distal epithelial cells of the mouse lung during development and in murine emphysema models [68]. However, except for the function as the Wnt signaling pathway inhibitors, recent studies showed that SFRPs are not merely Wnt-binding proteins, but can also antagonize different SFRPs' activity, interfere with BMP signaling by acting as proteinase inhibitors, decrease susceptibility to UV-induced apoptosis in primary culture of canine mammary gland tumors by NF-κB activation or JNK suppression, and interact with other receptors or matrix molecules [69]–[74]. Because of the limitation of the primary human airway epithelial cells, which prohibits us from performing the functional assays of the Wnt signaling pathway in a condition similar to that *in vivo*, we could not be certain that the down-regulation of Wnt signaling in healthy smokers and COPD patients is the result of SFRP2 up-regulation, despite the fact that SFRP2 could decrease WNT1-induced activation of Wnt reporter luciferase activity in HEK293 cells (p<0.05, Figure S3). Another limitation of this study is, although the down-regulation of the Wnt signaling pathway might induce the human airway epithelial cells to differentiate in healthy smokers and COPD patients, in the current study, we found there were decreased ciliated cells and increased undifferentiated cells (Table 1). There are a number of reasons for this discrepancy. Although the down-regulation of the Wnt signaling pathway could promote the repair and differentiation of airway epithelial cells in healthy smokers and smokers with COPD, the effect of smoking undoubtedly has a complex effect on multiple pathways. The confirmation of this concept is limited by the lack of a proper *in vitro* model. Furthermore, because the airway epithelial cells *in vivo* are composed a mixed populations including ciliated cells, secretory cells, basal cells and other cell populations such as clara cells, the microarray data may solely reflect changes in cell populations from patient to patient. However, it is unlikely because of the up-regulation of SFRP2 (which is specifically expressed in ciliated cells) in healthy smokers and smokers with COPD as well as the fact that the healthy smokers and smokers with COPD have lower percentage of ciliated cells.

Our findings suggest there is a complex Wnt signaling system in the adult human airway epithelium, which is subjected to a tightly regulated pattern that can respond to stress. Together, the data show that smoking induces down-regulation of Wnt signaling pathway, consistent with the concept that in response to the stress of cigarette smoking, the small airway epithelium Wnt pathway is suppressed, allowing for epithelial repair and differentiation.

## Supporting Information

Table S1 Expression of WNT Pathway Genes and Target Genes in Small Airway.
(0.12 MB DOC)

Figure S1 Ingenuity Pathway Analysis generated WNT/β-catenin gene network. A. IPA gene network of WNT/β-catenin signaling pathway genes that displayed 1.5-fold or greater changes between healthy smokers and healthy nonsmokers. B. IPA gene network of WNT/β-catenin signaling pathway genes that displayed 1.5-fold or greater changes between smokers with COPD and healthy nonsmokers. Additional information about the genes and the indicated interactions can be found at www.ingenuity.com.
(1.79 MB TIF)

Figure S2 TaqMan Real-time PCR confirmation of down-regulation of selected Wnt genes and Wnt target genes in healthy nonsmokers (NS), healthy smokers (S), and smokers with COPD (COPD). Each bar represents mean expression with standard error; p values are represented in brackets above the bars. The average value of healthy nonsmokers is determined as the calibrator for each gene.
(0.43 MB TIF)

Figure S3 The Wnt reporter assay in HEK293 cells. HEK293 cells were transiently transfected with Wnt reporter (Topflash) or Wnt reporter control (Fopflash) constructs, and stimulated with WNT1 plasmid or WNT1 plus SFRP2 plasmids, as indicated. The relative luciferase activity is plotted. The experiment was repeated three times and representative results are presented as mean ± standard deviation.
(0.40 MB TIF)
