# Supplementary material for: Down-Regulation of the Canonical Wnt β-Catenin Pathway in the Airway Epithelium of Healthy Smokers and Smokers with COPD
Source: PLoS One. 2011 Apr 7;6(4):e14793. doi: 10.1371/journal.pone.0014793 (PMC3072378; doi:10.1371/journal.pone.0014793)
Supplement: Table S1 — Expression of WNT Pathway Genes and Target Genes in Small Airway. (0.12 MB DOC) [file pone.0014793.s001.doc]

| **Gene symbol** | **Gene title** | **Probeset ID** | **Nonsmoker p call %** | **Smoker**  **p call %** | **COPD smoker**  **p call %** | **Smoker/**  **nonsmoker** | **Smoker/ nonsmoker p value** | **COPD/ nonsmoker** | **COPD/ nonsmoker p value** |
| --- | --- | --- | --- | --- | --- | --- | --- | --- | --- |
|  |  |  |  |  |  |  |  |  |  |
| WNT3 | wingless-type MMTV integration site family, member 3 | 224489_at | 36 | 28 | 32 | -1.06 | NS | 1.17 | NS |
| WNT4 | wingless-type MMTV integration site family, member 4 | 208606_s_at | 83 | 66 | 59 | -1.2 | NS | -1.30 | NS |
| WNT7B | wingless-type MMTV integration site family, member 7B | 217681_at | 43 | 26 | 36 | -1.14 | NS | -1.16 | NS |
| WNT9A | wingless-type MMTV integration site family, member 9A | 230643_at | 100 | 100 | 100 | 1.06 | NS | 1.23 | NS |
| WNT10A | wingless-type MMTV integration site family, member 10A | 223709_s_at | 43 | 36 | 32 | 1.08 | NS | 1.17 | NS |
| FZD1 | frizzled homolog 1 (Drosophila) | 204451_at | 98 | 93 | 100 | -1.17 | <0.01 | -1.13 | NS |
| FZD3 | frizzled homolog 3 (Drosophila) | 219683_at | 100 | 100 | 100 | -1.19 | NS | -1.26 | NS |
| FZD4 | frizzled homolog 4 (Drosophila) | 218665_at | 100 | 97 | 100 | -1.15 | <0.05 | -1.23 | <0.001 |
| FZD5 | frizzled homolog 5 (Drosophila) | 221245_s_at | 100 | 100 | 100 | 1.04 | NS | 1.23 | <0.01 |
| FZD6 | frizzled homolog 6 (Drosophila) | 203987_at | 100 | 100 | 100 | -1.14 | <0.05 | -1.16 | NS |
| FZD7 | frizzled homolog 7 (Drosophila) | 203705_s_at | 60 | 78 | 64 | 1.37 | <0.01 | 1.15 | NS |
| FZD8 | frizzled homolog 8 (Drosophila) | 224325_at | 100 | 95 | 91 | -1.54 | <0.0001 | -1.41 | <0.01 |
| LRP5 | low density lipoprotein receptor-related protein 5 | 209468_at | 66 | 83 | 91 | -1.04 | NS | 1.02 | NS |
| LRP6 | low density lipoprotein receptor-related protein 6 | 34697_at | 81 | 86 | 73 | 1.19 | <0.05 | 1.15 | NS |
| SFRP2 | secreted frizzled-related protein 2 | 223122_s_at | 11 | 81 | 91 | 4.28 | <0.0001 | 4.93 | <0.0001 |
| DKK1 | dickkopf homolog 1 (Xenopus laevis) | 204602_at | 47 | 43 | 59 | -1.03 | NS | 1.28 | NS |
| DKK3 | dickkopf homolog 3 (Xenopus laevis) | 202196_s_at | 21 | 36 | 23 | 1.26 | NS | 1.36 | NS |
| DKK4 | dickkopf homolog 4 (Xenopus laevis) | 231818_x_at | 89 | 84 | 95 | 1.09 | NS | 1.16 | NS |
| GSK3B | glycogen synthase kinase 3 beta | 209945_s_at | 100 | 100 | 100 | 1.03 | NS | 1.00 | NS |
| FRAT1 | frequently rearranged in advanced T-cell lymphomas | 219889_at | 100 | 100 | 100 | -1.22 | <0.01 | -1.14 | NS |
| FRAT2 | frequently rearranged in advanced T-cell lymphomas 2 | 209864_at | 100 | 100 | 100 | -1.34 | <0.0001 | -1.22 | <0.05 |
| DVL1 | dishevelled, dsh homolog 1 (Drosophila) | 203230_at | 100 | 100 | 100 | -1.17 | <0.05 | -1.04 | NS |
| DVL2 | dishevelled, dsh homolog 2 (Drosophila) | 57532_at | 100 | 100 | 100 | -1.07 | NS | -1.02 | NS |
| DVL3 | dishevelled, dsh homolog 3 (Drosophila) | 201908_at | 100 | 100 | 100 | -1.24 | <0.001 | -1.18 | <0.05 |
| CTNNB1 | catenin (cadherin-associated protein), beta 1, 88kDa | 1554411_at | 96 | 88 | 64 | -1.46 | <0.05 | -1.84 | <0.05 |
| APC | adenomatous polyposis coli | 203525_s_at | 100 | 100 | 100 | -1.13 | NS | -1.07 | NS |
| AEC | amino-terminal enhancer of split | 217729_s_at | 100 | 100 | 100 | -1.2 | NS | -1.11 | NS |
| AXIN1 | axin 1 | 212849_at | 34 | 22 | 23 | -1.16 | NS | -1.19 | NS |
| AXIN2 | axin 2 | 222696_at | 100 | 97 | 100 | -1.16 | <0.05 | -1.22 | <0.01 |
| LEF1 | lymphoid enhancer-binding factor 1 | 221558_s_at | 100 | 100 | 100 | -1.10 | NS | -1.09 | NS |
| TCF-7 | transcription factor 7 | 205255_x_at | 100 | 100 | 100 | -1.04 | NS | 1.01 | NS |
| TCF7L1 | transcription factor 7-like 1 (T-cell specific, HMG-box) | 221016_s_at | 100 | 100 | 100 | -1.71 | <0.0001 | -1.72 | <0.0001 |
| TCF7L2 | transcription factor 7-like 2 (T-cell specific, HMG-box) | 212759_s_at | 81 | 83 | 91 | -1.03 | NS | 1.26 | NS |
| MMP2 | matrix metallopeptidase 2 (gelatinase A, 72kDa gelatinase, 72kDa type IV collagenase) | 201069_at | 53 | 67 | 59 | 1.25 | <0.01 | 1.28 | NS |
| CDH1 | cadherin 1, type 1, E-cadherin (epithelial) | 201131_s_at | 100 | 100 | 100 | -1.25 | <0.001 | -1.14 | NS |
| ID2 | inhibitor of DNA binding 2, dominant negative helix-loop-helix protein | 201565_s_at | 100 | 100 | 100 | -1.16 | <0.05 | -1.12 | NS |
| GJA1 | gap junction protein, alpha 1, 43kDa | 201667_at | 91 | 88 | 73 | -1.61 | NS | -1.59 | NS |
| MYC | v-myc myelocytomatosis viral oncogene homolog (avian) | 202431_s_at | 85 | 83 | 86 | -1.19 | NS | -1.07 | NS |
| SOX9 | SRY (sex determining region Y)-box 9 | 202936_s_at | 100 | 100 | 100 | -1.88 | <0.0001 | -2.29 | <0.0001 |
| CCNA2 | cyclin A2 | 203418_at | 38 | 31 | 23 | -1.15 | NS | -1.12 | NS |
| MMP9 | matrix metallopeptidase 9 (gelatinase B, 92kDa gelatinase, 92kDa type IV collagenase) | 203936_s_at | 96 | 81 | 100 | -1.75 | NS | -1.12 | NS |
| MMP7 | matrix metallopeptidase 7 (matrilysin, uterine) | 204259_at | 47 | 14 | 9 | -1.66 | <0.01 | -2.62 | <0.0001 |
| ISL1 | ISL LIM homeobox 1 | 206104_at | 91 | 93 | 100 | -1.13 | NS | -1.07 | NS |
| CCND1 | cyclin D1 | 208712_at | 100 | 100 | 100 | -1.34 | <0.0001 | -1.39 | <0.001 |
| JAG1 | jagged 1 | 209099_x_at | 100 | 100 | 100 | -1.33 | <0.0001 | -1.32 | <0.01 |
| VEGFA | vascular endothelial growth factor A | 210512_s_at | 100 | 100 | 100 | -1.58 | <0.05 | -1.17 | NS |
| PLAUR | plasminogen activator, urokinase receptor | 210845_s_at | 100 | 97 | 100 | -1.02 | NS | 1.51 | <0.05 |
| TIAM1 | T-cell lymphoma invasion and metastasis 1 | 213135_at | 100 | 100 | 100 | -1.16 | NS | 1.19 | NS |
| JUN | Jun oncogene, mRNA (cDNA clone IMAGE:3947905) | 213281_at | 91 | 97 | 95 | 1.04 | NS | 1.32 | <0.05 |
| TWIST1 | twist homolog 1 (Drosophila) | 213943_at | 66 | 50 | 86 | 1.01 | NS | 1.31 | <0.05 |
| SFTPC | surfactant protein C | 215454_x_at | 30 | 24 | 41 | -1.19 | NS | 1.57 | NS |
| CLDN1 | claudin 1 | 218182_s_at | 98 | 97 | 86 | -1.37 | <0.0001 | -1.7 | <0.0001 |
| GREM1 | gremlin 1, cysteine knot superfamily, homolog (Xenopus laevis) | 218468_s_at | 53 | 38 | 32 | -1.32 | NS | -1.92 | <0.05 |
| NANOG | Nanog homeobox | 220184_at | 72 | 74 | 73 | 1.01 | NS | -1.19 | NS |
| STRA6 | stimulated by retinoic acid gene 6 homolog (mouse) | 221701_s_at | 89 | 97 | 95 | 1.29 | <0.001 | 1.33 | <0.01 |
| EDN1 | endothelin 1 | 222802_at | 74 | 76 | 77 | -1.10 | NS | -1.01 | NS |
| RHOU | ras homolog gene family, member U | 223168_at | 100 | 100 | 100 | -1.60 | <0.0001 | -1.88 | <0.0001 |
| SOX2 | SRY (sex determining region Y)-box 2 | 228038_at | 100 | 100 | 100 | -1.12 | <0.05 | -1.07 | NS |
| RUNX2 | runt-related transcription factor 2 | 232231_at | 100 | 100 | 100 | -1.51 | <0.0001 | -1.32 | <0.01 |
| PPARD | peroxisome proliferator-activated receptor delta | 37152_at | 100 | 98 | 100 | -1.01 | NS | 1.13 | NS |

1. NS, not significant
