# Supplementary figures and images for: Down-Regulation of the Canonical Wnt β-Catenin Pathway in the Airway Epithelium of Healthy Smokers and Smokers with COPD
Source: PLoS One. 2011 Apr 7;6(4):e14793. doi: 10.1371/journal.pone.0014793 (PMC3072378; doi:10.1371/journal.pone.0014793)

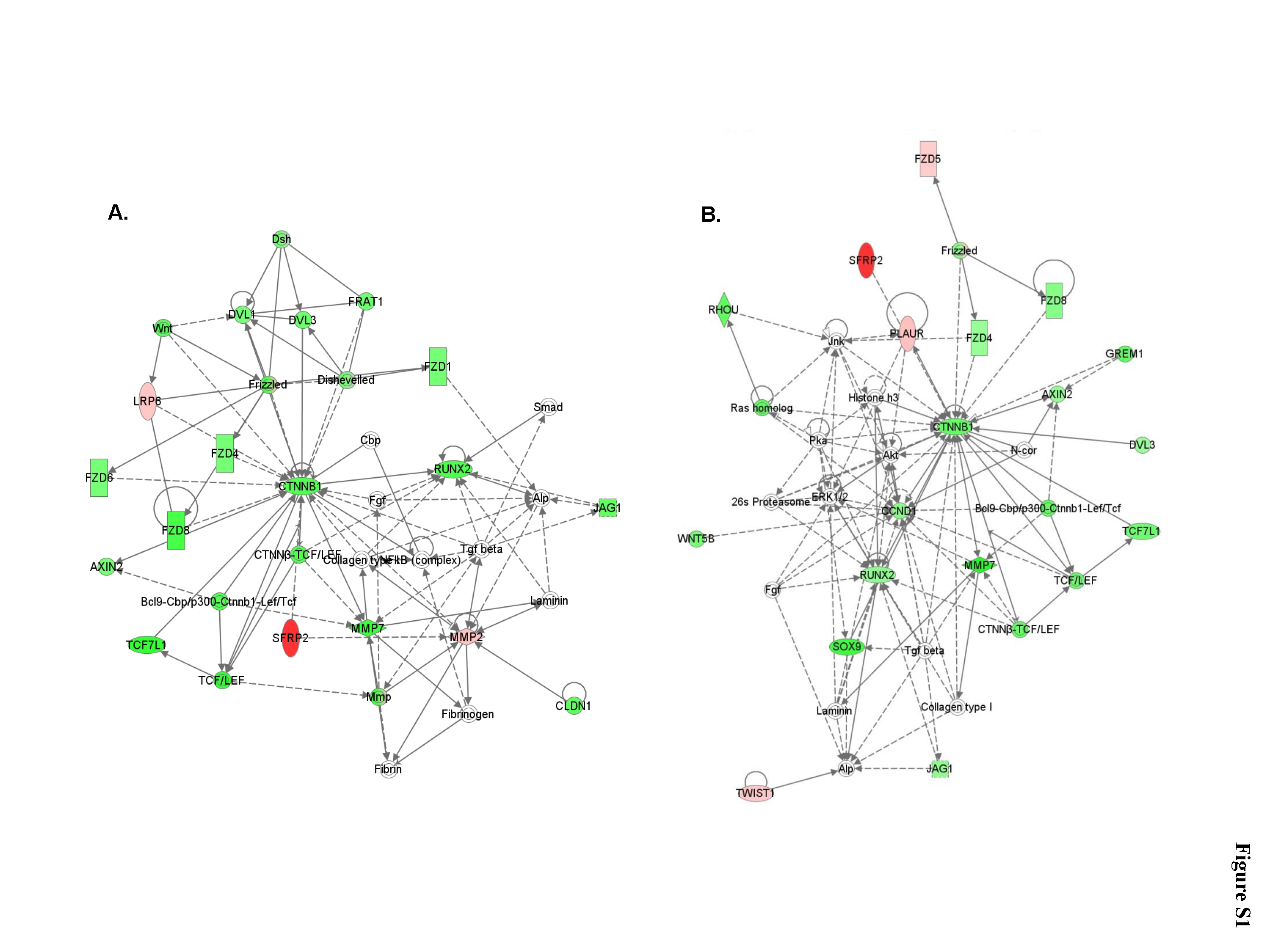

Supplement: Figure S1 — Ingenuity Pathway Analysis generated WNT/β-catenin gene network. A. IPA gene network of WNT/β-catenin signaling pathway genes that displayed 1.5-fold or greater changes between healthy smokers and healthy nonsmokers. B. IPA gene network of WNT/β-catenin signaling pathway genes that displayed 1.5-fold or greater changes between smokers with COPD and healthy nonsmokers. Additional information about the genes and the indicated interactions can be found at www.ingenuity.com. (1.79 MB TIF) [file pone.0014793.s002.tif]

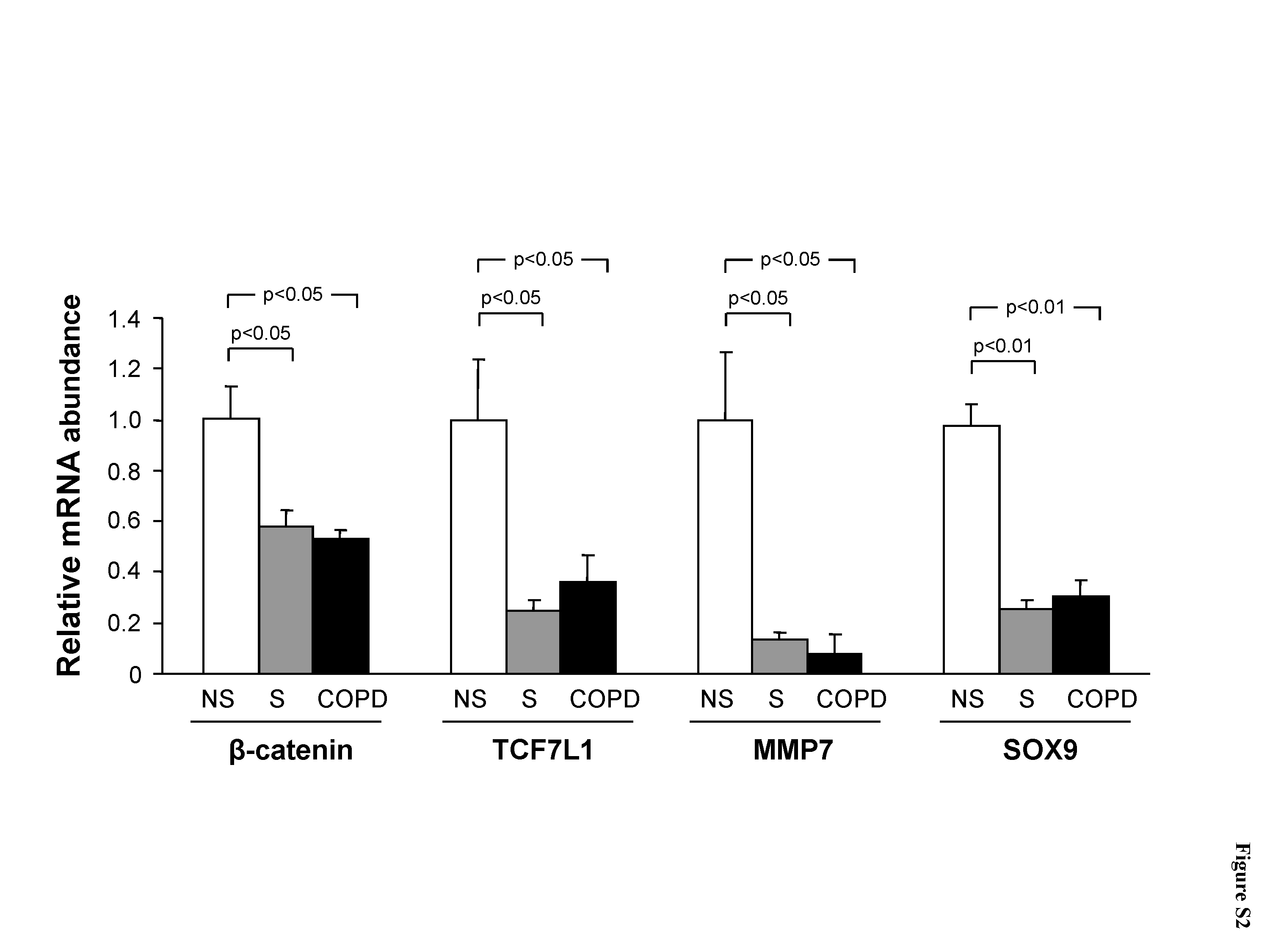

Supplement: Figure S2 — TaqMan Real-time PCR confirmation of down-regulation of selected Wnt genes and Wnt target genes in healthy nonsmokers (NS), healthy smokers (S), and smokers with COPD (COPD). Each bar represents mean expression with standard error; p values are represented in brackets above the bars. The average value of healthy nonsmokers is determined as the calibrator for each gene. (0.43 MB TIF) [file pone.0014793.s003.tif]

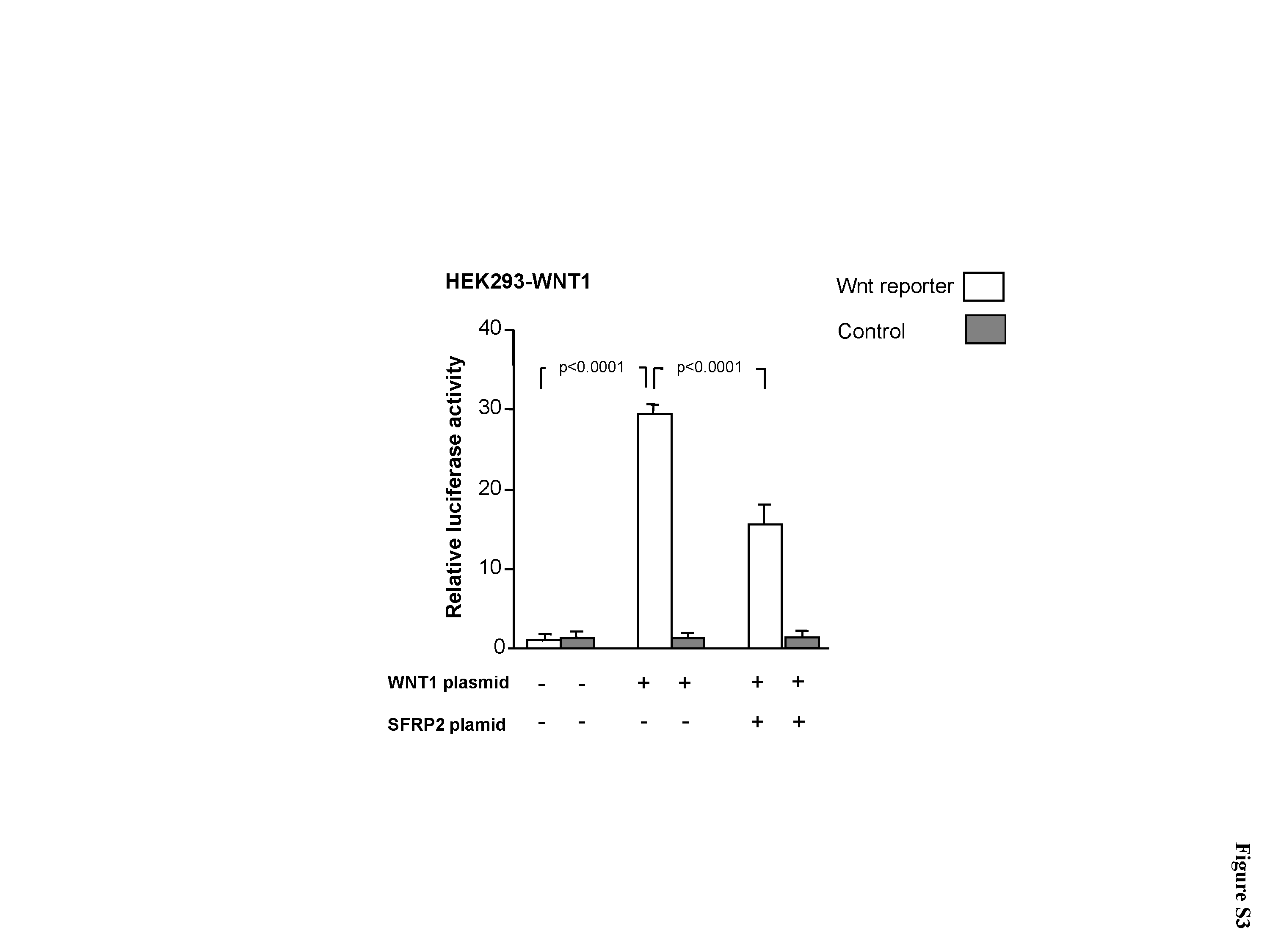

Supplement: Figure S3 — The Wnt reporter assay in HEK293 cells. HEK293 cells were transiently transfected with Wnt reporter (Topflash) or Wnt reporter control (Fopflash) constructs, and stimulated with WNT1 plasmid or WNT1 plus SFRP2 plasmids, as indicated. The relative luciferase activity is plotted. The experiment was repeated three times and representative results are presented as mean ± standard deviation. (0.40 MB TIF) [file pone.0014793.s004.tif]
